# Rumen Bacterial Community Composition in Holstein and Jersey Cows Is Different under Same Dietary Condition and Is Not Affected by Sampling Method

**DOI:** 10.3389/fmicb.2016.01206

**Published:** 2016-08-03

**Authors:** Henry A. Paz, Christopher L. Anderson, Makala J. Muller, Paul J. Kononoff, Samodha C. Fernando

**Affiliations:** ^1^Department of Animal Science, University of Nebraska-Lincoln, LincolnNE, USA; ^2^School of Biological Sciences, University of Nebraska-Lincoln, LincolnNE, USA; ^3^Department of Food Science and Technology, University of Nebraska-Lincoln, LincolnNE, USA

**Keywords:** Holstein cow, Jersey cow, rumen bacterial community, rumen sample, esophageal tubing, rumen cannula

## Abstract

The rumen microbial community in dairy cows plays a critical role in efficient milk production. However, there is a lack of data comparing the composition of the rumen bacterial community of the main dairy breeds. This study utilizes 16S rRNA gene sequencing to describe the rumen bacterial community composition in Holstein and Jersey cows fed the same diet by sampling the rumen microbiota via the rumen cannula (Holstein cows) or esophageal tubing (both Holstein and Jersey cows). After collection of the rumen sample via esophageal tubing, particles attached to the strainer were added to the sample to ensure representative sampling of both the liquid and solid fraction of the rumen contents. Alpha diversity metrics, Chao1 and observed OTUs estimates, displayed higher (*P* = 0.02) bacterial richness in Holstein compared to Jersey cows and no difference (*P* > 0.70) in bacterial community richness due to sampling method. The principal coordinate analysis displayed distinct clustering of bacterial communities by breed suggesting that Holstein and Jersey cows harbor different rumen bacterial communities. Family level classification of most abundant (>1%) differential OTUs displayed that OTUs from the bacterial families *Lachnospiraceae* and p-2534-18B5 to be predominant in Holstein cows compared to Jersey cows. Additionally, OTUs belonging to family *Prevotellaceae* were differentially abundant in the two breeds. Overall, the results from this study suggest that the bacterial community between Holstein and Jersey cows differ and that esophageal tubing with collection of feed particles associated with the strainer provides a representative rumen sample similar to a sample collected via the rumen cannula. Thus, in future studies esophageal tubing with addition of retained particles can be used to collect rumen samples reducing the cost of cannulation and increasing the number of animals used in microbiome investigations, thus increasing the statistical power of rumen microbial community evaluations.

## Introduction

The US dairy cow herd is predominated by the Holstein breed followed by the Jersey breed representing 83% and 7% of the total population, respectively (Council on Dairy Cattle Breeding [CDCB], 2015). These breeds have been shown to be genetically differentiated at each chromosome (Melka and Schenkel, 2012). Marked production-related phenotype differences exist between Holstein and Jersey cattle, specifically higher milk yield in Holstein cows and higher milk protein and fat content in Jersey cows (Capper and Cady, 2012). The difference in nutrient density in the milk of these breeds influences the processed product yield. For instance, Cheddar cheese yield is 0.101 kg/kg of milk and 0.125 kg/kg of milk for Holstein and Jersey cows, respectively (Capper and Cady, 2012). Genetic differences between these two breeds do not fully explain their productive responses suggesting that other factors contribute to the differences between the two breeds. Heavier gastrointestinal tract as a proportion of body weight in Jersey compared to Holstein cows is a morphometric difference that has been associated with improved production efficiency in the Jersey cow (Beecher et al., 2014). One hypothesis is that the rumen microbiota composition between Holstein and Jersey cows differs and that microbial species diversity and distribution contributes towards production-related phenotypes. Similar to the microbial community in the gut of non-ruminants, the structure and function of the microbial community within the rumen of cows is shaped by the dynamic physical, chemical, and predatory environment. In turn, the microbial community regulates nutrient cycling to the host (Church, 1993). In the dairy cow, the rumen microbial community plays a critical role in volatile fatty acids production, B-vitamin synthesis, and microbial cell protein synthesis, which are critical for the animal’s well-being and efficient milk production (Krehbiel, 2014). Moreover, the structure of the bacterial community has been correlated with feed efficiency (Hernandez-Sanabria et al., 2012), milk yield, and milk composition (Jami et al., 2014; Lima et al., 2015). Despite production-related phenotypic differences between Holstein and Jersey breeds, previous studies have not evaluated the overall rumen microbiota composition of these two breeds. For example, Beecher et al. (Beecher et al., 2014) compared the two breeds using quantitative real-time PCR to evaluate abundance of two fiber digesting bacteria. The aforementioned authors reported a higher relative abundance of *Ruminococcus flavefaciens* in Holstein cows compared to Jersey cows and no difference in *Fibrobacter succinogenes*. However, a more in-depth comparison is warranted to improve the understanding of the difference in the rumen bacterial community composition in these two breeds.

In addition to the knowledge gap in bacterial community composition in Holstein and Jersey cows, the method of obtaining a representative rumen sample has been widely debated (Steiner et al., 2015). Studies have demonstrated bacterial species composition between the rumen liquid and solid phases to significantly differ (Cho et al., 2006; Sadet et al., 2007); suggesting that sampling method can greatly influence the microbial community composition being evaluated. Thus, collection of a representative rumen sample containing both solid and liquid fractions is critical to describe the microbial community composition. Two common methods widely used to sample whole rumen contents are either through a rumen cannula or through esophageal tubing of the animal (Duffield et al., 2004; Terré et al., 2013). Sampling through a rumen cannula is direct and allows for the collection of a representative sample of rumen contents, but it is an invasive method and the costs related to the surgical procedure and animal care limits the number of animals that can be cannulated and used in a study. Esophageal tubing is less invasive, cheaper, and can be used to sample a large group of animals, thus increasing statistical power of the experiment. Concerns related to inconsistent positioning of the tube in the rumen, saliva contamination of the sample, and inconsistent recovery of both liquid and solid phases (Duffield et al., 2004; Shen et al., 2012) have limited the use of esophageal tubing for rumen microbial ecology investigations. However, studies have concluded that representative rumen samples are collected using esophageal tubing when evaluating fermentation parameters (Shen et al., 2012; Ramos-Morales et al., 2014). To better assess esophageal tubing as a method to sample rumen contents, a comparison of the rumen bacterial species composition of the same group of animals sampled both through the rumen cannula and esophageal tubing is needed. Therefore, this study utilizes 16S rRNA gene sequencing as a first step towards describing the rumen bacterial community composition in Holstein and Jersey cows under same dietary condition and additionally investigates the effect of sampling method on rumen bacterial community composition.

## Materials and Methods

### Animals and Diet

Animal care and experimental procedures were conducted according to the guidelines of the University of Nebraska-Lincoln (UNL) Animal Care and Use Committee. Five ruminally cannulated Holstein lactating cows averaging 6.2 ± 0.70 (mean ± SE) years of age (range = 3.9 years) and four Jersey lactating cows (not cannulated) averaging 4.5 ± 0.39 years of age (range = 1.9 years) were used in the experiment. All Holstein cows had been part of the UNL dairy herd throughout their lives, whereas Jersey cows were purchased from a commercial dairy farm and had been part of the UNL dairy herd for 230 days under the same management as the Holstein cows before the start of the experiment. Cows were housed in a temperature-controlled room in individual tie stalls, and were fed the same diet once daily at 1000 h for ad libitum consumption at 110% of expected intake for four weeks. On a dry matter (DM) basis, the diet was comprised of 51% forage and 49% concentrate and was formulated to meet or exceed the requirements of a lactating cow (Table S1).

### Rumen Content Sampling

On the 28th day of the feeding regimen, a single rumen sample (both solid and liquid fractions) from each Holstein and Jersey cow was collected at 1300 h via esophageal tubing. The esophageal tubing apparatus was prepared by coupling one end of the esophageal tube to a metal strainer as described by Raun and Burroughs (1962) and the other end was connected to a 125-mL Nalgene bottle (Thermo Scientific Inc., Waltham, MA, USA) using a “Tee” connection. The remaining end of the “Tee” was connected to a Masterflex vacuum pump (model 7015-10; Cole Parmer, Vernon Hills, IL) (Figure S1). Rumen samples were collected by passing the tube containing the metal strainer through a Frick speculum into the ventral sac. The first 200-mL of rumen fluid were discarded to minimize saliva contamination. Then 40-mL of rumen fluid were collected and placed into a 50-mL propylene conical tube (Thermo Scientific Inc., Waltham, MA, USA). After removal of the esophageal tube, particles attached to the metal strainer were recovered and added into the conical tube to collect a sample more adequately representative of the rumen content and then samples were snap-frozen in liquid nitrogen. Across samples, particles ranged from 10 to 15% of the total sample. Between sampling, the metal strainer and Frick speculum were thoroughly washed with warm water and water was run through the stomach tube to prevent cross contamination from the previous animal. Additionally, the removal of the first 200-mL also prevented any cross contamination.

Immediately after the collection of the esophageal sampling, another sample was collected from the Holstein cows via the rumen cannula. Ruminal contents were collected from the dorsal, ventral, and caudal areas of the rumen. The digesta were mixed and a representative sample was collected and snap-frozen in liquid nitrogen for bacterial community analysis. All samples collected via esophageal tubing or via the cannula were stored in an -80°C freezer until used for DNA extraction and bacterial community analysis.

### DNA Isolation, 16S rRNA Library Preparation, and Sequencing of the V3 Region

DNA was extracted using the PowerMag^TM^ Soil DNA Isolation Kit (Mo Bio Laboratories, Inc., Carlsbad, CA, USA) according to the manufacturer’s protocol with the following modifications: the two bead-beating steps were performed in a TissueLyser (QIAGEN Inc., Valencia, CA, USA) and samples were incubated in a 95°C water bath for 5 min between bead-beading steps. The resulting DNA was evaluated for DNA quality and concentration and was used for tag sequencing of the V3 region of the 16S rRNA gene. The V3 region of the 16S rRNA gene specific to eubacterial communities was amplified using universal primers 341F and 518R (barcoded) (Whiteley et al., 2012). The PCR reactions were performed in 25 μL volumes and contained 0.5 Units of Terra DNA polymerase (Clontech Laboratories, Mountain view, CA, USA), 1X reaction buffer, 200 μM dNTPs (Invitrogen, Carlsbad, CA, USA), 200 nM of each primer, and 20-50 ng of nucleic acid template or no-template control. The cycling conditions were an initial denaturation of 98°C for 3 min, followed by 25 cycles of 98°C for 30 s, 52°C for 30 s, and 68°C for 40 s; and a final extension of 68°C for 4 min. Following amplification, PCR products were analyzed on a 1.8% agarose gel to confirm correct product size. Normalized amplicons (1-2 ng/μL) from 96 samples were pooled together using an epMotion M5073 liquid handler (Eppendorf AG, Hamburg, Germany). Pooled libraries were sequenced on an Ion Torrent^TM^ Personal Genome Machine (PGM; Life Technologies, Carlsbad, CA, USA) using the 200 bp Sequencing Kit v2 on a 316 chip according to manufacturer’s protocols. The methods used for emulsion PCR, bead deposition, and sequencing on the PGM were as described by the manufacturer. Sequence data were deposited in the NCBI Sequence Read Archive (SRA) under the accession no. SRP071307.

### Data Processing and Bacterial Community Analysis

Detailed information about the bioinformatics pipeline that contains the mapping file, all the scripts and commands, and an R Markdown (dairy_breeds.Rmd) and rendered R Markdown (dairy_breeds.html) files were deposited at https://github.com/FernandoLab/2016_Paz_et_al_Dairy_Breeds. The R Markdown file allows to fully reproduce the analyses used in this experiment. Initial quality control of the generated sequences was performed using the Torrent Suite Software version 3.6.2, which included trimming of the 3′ end of sequences that dropped below the average Q15 score over a 30 bp window and removing sequences with unidentified bases (N). Resulting sequences were downloaded from the Torrent Suite and demultiplexed using the QIIME software package (version 1.9.1) (Caporaso et al., 2010). During demultiplexing, sequences with an average quality score <25 during were removed. Following demultiplexing, universal primers used for sequencing were removed, allowing 1 mismatch in the 5’ (518R) primer and 2 in the 3′ reverse primer (341F). Sequences shorter than 130 bp were removed and remaining sequences were trimmed to a fixed length of 130 bp. Quality trimmed sequences were then reverse complemented, screened for chimeric sequences using UCHIME (Edgar et al., 2011), and pre-clustered using the pseudo-single linkage-clustering algorithm to remove reads that resulted from sequencing errors (Huse et al., 2010). Subsequently, sequences were binned into operational taxonomic units (OTUs) at 97% similarity using the UPARSE pipeline (USEARCH v7.0.1090) (Edgar, 2013). Representative sequences from each OTU were assigned taxonomy by using the UCLUST consensus taxonomy assigner (QIIME default) method with Greengenes database release 119 (McDonald et al., 2012) as reference sequences.

A core measurable microbiome was determined based on breed (Holstein vs Jersey) and rumen sampling method (rumen cannula vs esophageal tubing) and was defined as OTUs present in at least 80% of the Holstein cows (4/5 cows) and 75% of the Jersey cows (3/4 cows).

### Statistical Analyses

The OTU table was rarefied across samples to the lowest sample depth (12,141 reads) using QIIME based on the Mersenne Twister pseudorandom number generator. All statistical analyses were performed with samples at an even depth. Alpha diversity estimators Chao1 and observed OTUs and rarefaction curves were calculated for the overall bacterial community using QIIME. Good’s coverage test was performed to evaluate if adequate sampling depth was achieved. Mean alpha diversity estimates for both breed and rumen sampling method were compared using the two-sided t-test in R (R Core Team, 2014). For the core bacterial community, the weighted UniFrac distance matrix was calculated using QIIME. Even depth across samples avoided biases that could be encounter when using the Unifrac metric (Lozupone et al., 2011). Core bacterial community composition differences were evaluated using the weighted UniFrac distance matrix as an input for a permutational multivariate analysis of variance (PERMANOVA) in R using the vegan package (adonis function) (Oksanen et al., 2015) where breed or sampling method were used as main effects. In addition, the weighted UniFrac distance matrix was used in the principal coordinate analysis (PCoA) to visualize relationships between samples. Homogeneity of molecular variance (HOMOVA) was determined using mothur (Schloss et al., 2009). Significance was declared at *P* ≤ 0.05 throughout this study. The linear discriminant analysis effect size (LEfSe) (Segata et al., 2011) was used to identify specific OTUs that differed between breeds and sampling methods. LEfSe uses a non-parametric factorial Kruskal-Wallis sum-rank test followed by a linear discriminate analysis to identify both statistically significant and biological relevant features. The core OTUs relative abundances was used as an input for LEfSe.

Venn diagrams were constructed in R using the venn function in the gplots package of R (Warnes et al., 2015). Heat maps were generated in R using the heatmap.2 function to display OTUs with a relative abundance greater than 1%. Bray-Curtis dissimilarity was used to compute the distance between samples and dendrograms were generated using average linkage hierarchical clustering.

## Results

### Lactation Responses

Holstein cows averaged 23.8 ± 0.58 kg/d dry matter intake (DMI) and 38.5 ± 3.55 kg/d milk yield, whereas Jersey cows averaged 19.2 ± 0.29 kg/d DMI and 23.8 ± 1.94 kg/d milk yield (Figure S2). This study was not designed to evaluate lactation responses between these two breeds, nevertheless, observed differences in DMI and milk yield were as commonly reported (NRC, 2001).

### Richness, Diversity Estimates, and Rumen Bacteria Composition

As described in the statistical analyses section, samples were rarefied to an even depth (12,141 reads). At this even depth across samples, 3000 OTUs were generated. To assess if the sampling depth was adequate to evaluate rumen bacterial composition, rarefaction curves were generated using Chao1 and observed OTUs for each breed and sampling method (Figure S3). Rarefaction curves for both breed and sampling method did not converge but showed a diminishing rate of new OTU identification as the number of reads per sample increased, implying that sampling depth was adequate for evaluating dominant members of the rumen bacterial community. The Good’s coverage test showed the sequencing depth was able to characterize ≥95.7% of the bacterial community. Alpha diversity metrics, Chao1 and observed OTUs estimates, displayed higher (*P* = 0.02) bacterial richness in Holstein compared to Jersey cows (Table S2). Mean Chao1 values were 1,846 and 1,552 and observed OTUs were 1,343 and 1,172 for Holstein and Jersey cows, respectively. Alpha diversity metrics were similar (*P* > 0.70) for the samples from Holstein cows collected from the rumen cannula and esophageal tubing.

**FIGURE 1:**
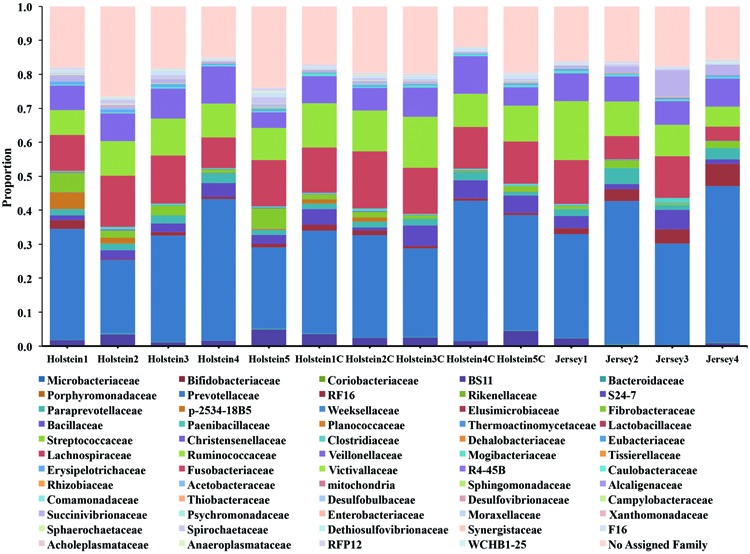
**Family level classification of the bacterial community composition across breeds and sampling methods.** Samples labeled with a C at the end were collected via rumen cannula, whereas rest of the samples were collected via esophageal tubing.

The taxonomic analysis of the reads revealed the presence of 4 main phyla (relative abundance >1%) in the rumen of Holstein and Jersey cows regardless of sampling method (Figure S4). *Bacteroidetes* and *Firmicutes* were the predominant phyla accounting for 51.4 and 40.4% of the total reads, respectively, followed by *Proteobacteria* and *Fibrobacteres* accounting for 2.5 and 2.1% of the total reads, respectively. At the family level, 81.8% of the total reads were annotated and the taxonomic analysis revealed the presence of 3 main families in the rumen of Holstein and Jersey cows regardless of sampling method. *Prevotellaceae*, *Lachnospiraceae*, and *Ruminococcaceae* were the predominant families accounting for 32.9, 11.9, and 10.7% of the total reads, respectively (**Figure 1**). To reduce animal-to-animal variation and to identify the core bacterial communities within the two different breeds, a core measureable microbiome was estimated (see Materials and Methods). The core measureable microbiome contained 92.6% of the rarefied quality filtered sequences (157,339/169,974 rarefied quality filtered sequences) and consisted of 487 OTUs for the Holstein cows and 473 OTUs for the Jersey cows (Figure S5). A total of 1747 OTUs were shared across the two breeds representing 94.8% of the sequences, whereas 2102 OTUs were shared between the two sampling methods representing 98.8% of the sequences.

### Differences in Bacterial Community Composition in Dairy Breeds and Sampling Method

**FIGURE 2:**
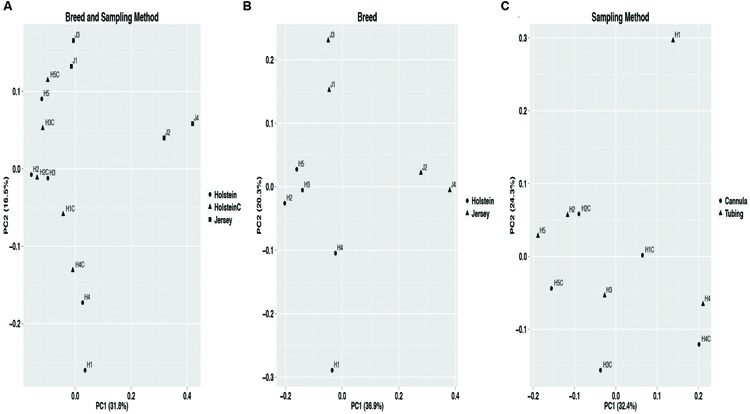
**Principal coordinate analysis (PCoA) using the weighted UniFrac distance matrix displaying significant structuring of bacterial communities by breed and not by sampling method.** The core OTUs data set (see Materials and Methods) was used for PCoA. **(A)** Full data set including sample collected through the rumen cannula (labeled with a C at the end) and esophageal tubing from both Holstein (H) and Jersey (J) cows, **(B)** bacterial communities for Holstein and Jersey cows collected via esophageal tubing (breed effect), and **(C)** bacterial communities for samples collected via esophageal tubing and via the rumen cannula from Holstein cows (sampling method effect).

The bacterial community composition differences by breed and sampling method were evaluated using PERMANOVA with the weighted UniFrac distance matrix. Breed displayed a significant (*P* = 0.02) effect on bacterial community composition, whereas sampling method had no effect (*P* = 0.62). Furthermore, the PCoA plot containing all samples displayed samples clustered in two groups by breed (**Figure 2**). This suggests sampling method (samples from Holstein cows collected via the rumen cannula or esophageal tubing) had no significant impact on the observed rumen bacterial community structure. Hierarchical clustering of all core OTUs with a relative abundance of >1% (Figure S6) supported that sampling method had no effect on bacterial community structure as samples from the cannula and esophageal tubing of Holstein cows clustered together. HOMOVA results showed that intra-breed (*P* = 0.25) and intra-method sample (*P* = 0.29) variation was not significant.

### OTU-Level Differences in Rumen Bacterial Community Composition by Breed

**FIGURE 3:**
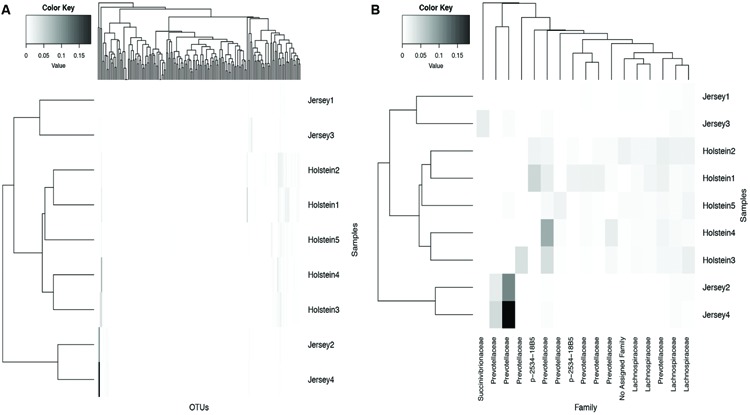
**Hierarchical clustering of core OTUs in Holstein and Jersey cows. (A)** All core OTUs that were significantly different between Holstein and Jersey cows (see Materials and Methods) and **(B)** OTUs that are significantly different between Holstein and Jersey cows with a minimum relative abundance of > 1%.

LEfSe identified 181 core OTUs to differ significantly (*P* < 0.05 and LDA > 2.0) between Holstein and Jersey cows. To visualize patterns in the differential OTUs among the two breeds, a heat map was generated that represents OTUs with a minimum relative abundance of 1% (**Figure 3B**). Family level classification of differential OTUs help identify that OTUs belonging to the *Lachnospiraceae* and p-2534-18B5 families were significantly higher in abundance in Holstein cows compared to Jersey cows. An OTU belonging to the family *Succinivibrionaceae* had higher abundance in Jersey cows compared to Holstein cows while both breeds had higher relative abundance of OTUs belonging to the family *Prevotellaceae*.

LEfSe identified 48 OTUs to differ significantly (*P* < 0.05 and LDA > 2.0) due to sampling method. Out of the 48 OTUs only five OTUs had a relative abundance higher than 1%. All of the five OTUs were higher in samples collected via esophageal tubing compared to rumen cannula and two belonged to the Fibrobacteraceae family while the others belonged to the Veillonellaceae, Prevotellaceae, and Lachnospiraceae families.

## Discussion

The microbial community in the rumen enables the dairy cow to harvest energy from fibrous, low quality feeds. This microbial community rapidly changes from birth to the time the cow reaches maturity, and is influenced by diet (McCann et al., 2014), feeding strategy (Golder et al., 2014), environment, age (Jami et al., 2013), feeding behavior (Prendiville et al., 2010), and host factors (Weimer et al., 2010). Thus, it is suggested that due to host genotype-microbiota interactions, different cattle breeds may carry different microbial species compositions. Despite the importance of genetic factors on microbial community composition and the marked production-related phenotypic differences between Holstein and Jersey cows, there is limited data comparing the overall bacterial species composition of these two breeds of dairy cattle. Therefore, the objectives of this study were to describe the rumen bacterial community composition of Holstein and Jersey cows under the same dietary and management conditions while also assessing the effect of sampling method (cannula and tubing) on the rumen bacterial community composition.

Alpha diversity metrics (Table S2) displayed a higher diversity in Holstein cows compared to Jersey cows regardless of sampling method. This suggests that each breed harbored a distinct bacterial community with respect to species composition and/or relative abundance. The distinct bacterial community structure observed in Holstein and Jersey cows were clearly seen in the PCoA plot (**Figure 2**), where bacterial communities clustered by breed. Life trajectory history (Ericsson et al., 2015), environmental interactions (Friswell et al., 2010), and genetic background (Deloris Alexander et al., 2006) have been shown to influence microbial composition in the gut. In the murine, genetic background was a greater determinant of the gut microbiota compared to sex (Kovacs et al., 2011). Moreover, studies (Benson et al., 2010; McKnite et al., 2012) have demonstrated that quantitative trait loci is linked to microbial taxa, providing evidence that host genotype can affect microbial community composition. Thus, observed bacterial community composition difference in this study may be in part a result of genomic differences in the two breeds. However, as a majority of sequences (94.8%) were shared between the two breeds suggesting that to a larger extent the microbial community of predominant bacterial species is similar. Hierarchal clustering of the predominant core OTUs (relative abundance >1%) (**Figure 3**) resulted in clustering of bacterial OTUs based on significant abundance in Holstein or Jersey cows, further suggesting that unique taxa and taxa with differential abundance is present within the two different breeds. However, the lower abundance of bacterial taxa identified as differential OTUs suggests that the significantly different bacterial OTUs detected may not be the major players in the rumen. The bacterial families *Lachnospiraceae* and P-2534-18B5 were predominant in Holstein cows compared to Jersey cows (**Figure 1**; Table S3). Additionally, multiple unclassified OTUs belonging to family *Prevotellaceae* were differentially abundant in the two different breeds. *Prevotella* sp. is a common organism found in the rumen of dairy heifers (Golder et al., 2014) and cows (de Menezes et al., 2011; Golder et al., 2014) under varying dietary regimes, therefore, the detection of different OTUS in the two breeds suggests that different strains or species of *Prevotella* are present in different relative abundances in the two breeds. The *Prevotellaceae* family belongs to the *Bacteroidetes* phylum and includes members with hemicellulolytic and proteolytic activity (Matsui et al., 2000), whereas the *Lachnospiraceae* family belongs to the *Firmicutes* phylum and include members with fibrolytic and cellulolytic activity (Thoetkiattikul et al., 2013). Overall, differences in abundance of OTUs from these families suggest varying cellulolytic activity between Holstein and Jersey cows. A study with a larger population size is needed to better understand the difference in the rumen bacterial community composition in these two breeds; however, this work clearly suggests that Holstein and Jersey cows harbor distinct bacterial communities. In addition, future research is needed to specifically evaluate if compositional differences in the rumen cellulolytic bacterial communities result in differences in production related measures.

Sampling through the rumen cannula is the standard method to collect rumen digesta samples for microbial community analysis, rumen pH, and volatile fatty acids analysis (Nocek, 1996). However, this procedure requires surgical intervention and is cost prohibitive to perform in larger sample population. Therefore, the approach of sampling through a rumen cannula limits the number of animals that can be used in a study and reduces the power of the experiment. However, as an alternative, rumen samples from more animals can be collected via esophageal tubing. Studies utilizing this approach instead of rumen cannula have demonstrated that samples collected through esophageal tubing can adequately represent rumen fermentation parameters such as pH, volatile fatty acids profile, and mineral concentrations (Shen et al., 2012; Terré et al., 2013; Ramos-Morales et al., 2014). However, the use of esophageal tubing for rumen microbial community analysis is less well adapted due to the belief that a representative sample cannot be collected through esophageal tubing. In the present study, we were able to collect a representative sample via esophageal tubing by ensuring the collection of feed particles retained in the metal strainer. Thus, collecting a representative rumen sample that contained both liquid and solid fractions. Furthermore, by discarding the first 200-mL of rumen fluid we greatly minimize the saliva contamination. It is important to note that animals must be restrained during rumen sample collection using esophageal tubing, thus staff experienced in the use of this procedure is important if repeated sampling throughout the day is planned both to ensure animal welfare and adequate sampling (Shen et al., 2012; Steiner et al., 2015).

The core measureable microbiome identified through 16S rRNA gene sequencing from the Holstein cows samples via the cannula and esophageal tubing were similar suggesting that sampling method did not significantly change bacterial community composition. This observation is in agreement with previous studies (Lodge-Ivey et al., 2009; Terré et al., 2013) using denaturing gradient gel electrophoresis, which reported no difference in the bacterial community composition from rumen samples taken via the rumen cannula or esophageal tubing. The esophageal tube device may favor the collection of a sample over-representing the liquid phase in comparison to the solid phase, therefore it is critical to collect the particles collected in the metal strainer to ensure the use of both liquid and solid fractions for DNA extraction. The addition of particles attached to the metal strainer resulted in a more representative sample as alpha and beta diversity estimates and LEfSe analysis supported consistent bacterial community composition between the two sampling methods.

Comprehensive knowledge of the bacterial community composition in dairy cows is important to understand the relationships between host and bacterial community and also to develop feeding strategies to positively influence feed efficiency and milk yield. This study describes the rumen bacterial community in Holstein and Jersey cows under same dietary and management conditions in an attempt to identify compositional changes in the rumen bacterial community that would describe the marked changes in production related measures. Additionally, this study demonstrates esophageal tubing can be used to collect representative rumen samples to evaluate rumen bacterial community structure, which provides the opportunity to evaluate bacterial community composition in larger groups of animals under normal production settings and to increase the number of animals used in studies, thus increasing the statistical power.

## Authors Contributions

HP, SF, PK, designed the research project and helped prepare the manuscript. HP and CA conducted the bioinformatics analysis. MM performed the laboratory analyses.

## Conflict of Interest Statement

The authors declare that the research was conducted in the absence of any commercial or financial relationships that could be construed as a potential conflict of interest.
