# Supplementary material for: Rumen Bacterial Community Composition in Holstein and Jersey Cows Is Different under Same Dietary Condition and Is Not Affected by Sampling Method
Source: Front Microbiol. 2016 Aug 3;7:1206. doi: 10.3389/fmicb.2016.01206 (PMC4971436; doi:10.3389/fmicb.2016.01206)
Supplement: Supplementary file 1 [file Data_Sheet_1.PDF]

Supplementary Material

Rumen bacterial community composition in Holstein and Jersey cows is different under same dietary condition and is not affected by sampling method

Henry A. Paz[1], Christopher L. Anderson[1,3], Makala J. Muller[1], Paul J. Kononoff[1], and Samodha C. Fernando[1,2,3*]

[1]Department of Animal Science, University of Nebraska-Lincoln, Lincoln, NE, USA

[2]Department of Food Science and Technology, University of Nebraska-Lincoln, Lincoln, NE, USA

[3]School of Biological Sciences, University of Nebraska-Lincoln, Lincoln, NE, USA

[*] **Correspondence:**
Samodha C. Fernando
sfernando2@unl.edu

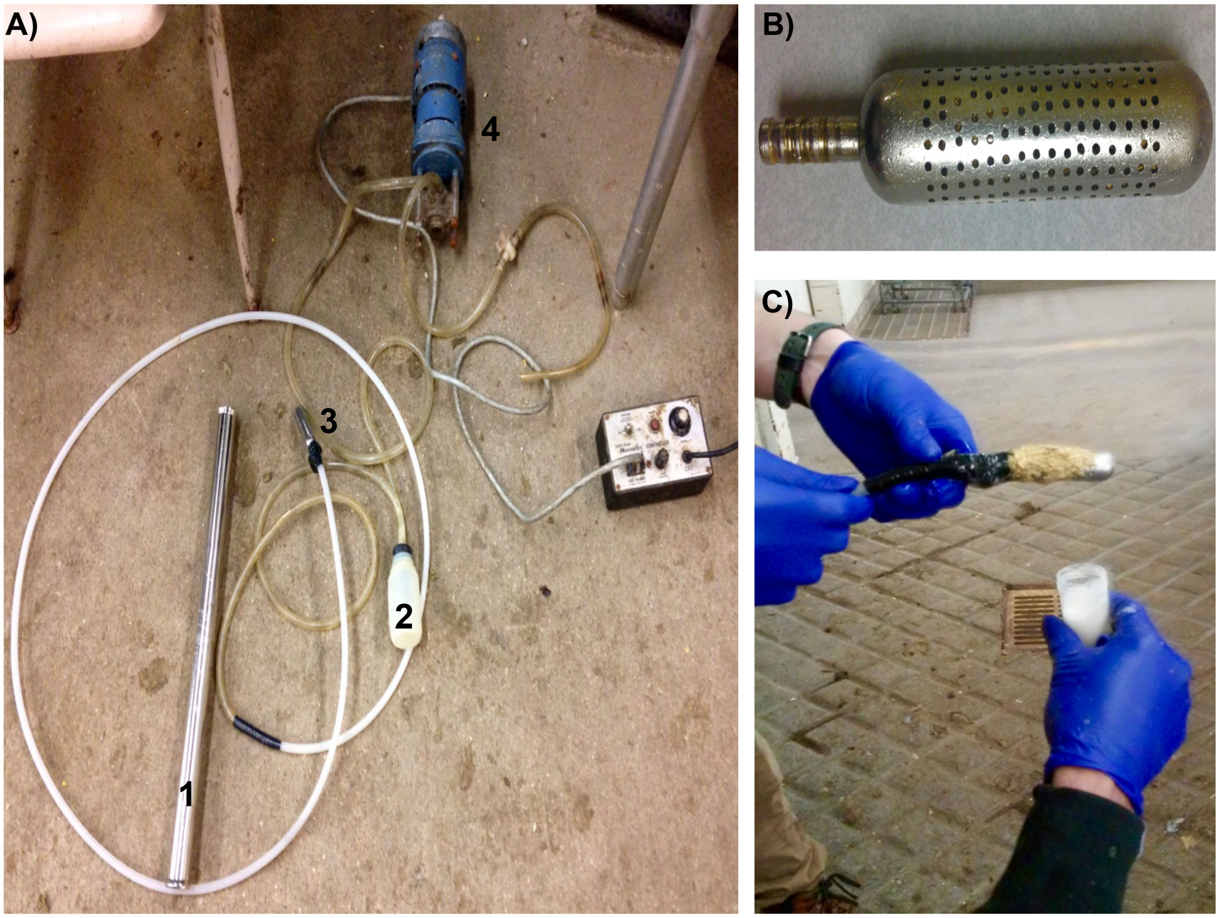

Supplementary Figure 1. Esophageal tubing apparatus components and sample collection. **(A)** Esophageal tubing apparatus: (1) Frick speculum, (2) 125-mL Nalgene bottle, (3) metal strainer, and (4) Masterflex vacuum pump, **(B)** metal strainer as described by Raun and Burroughs (1962)[a], **(C)** rumen content samples included particles attached to the metal strainer which ranged from 10 to 15% of the total sample.

[a] Raun, N. S., and Burroughs, W. (1962). Suction strainer technique in obtaining rumen fluid samples from intact lambs. *J. Anim. Sci.* 21, 454-457. doi: 10.2134/jas1962.213454x

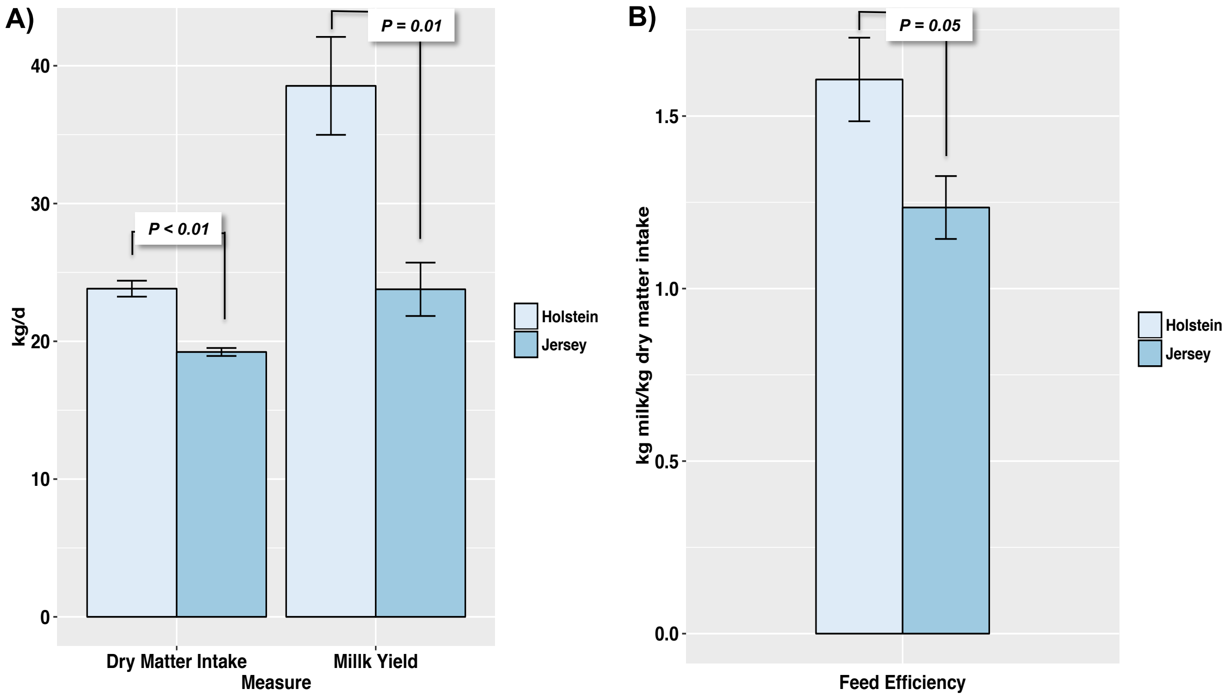

Supplementary Figure 2. Production responses from Holstein (n = 5) and Jersey (n = 4) cows. (A) Dry matter intake and milk yield, and **(B)** feed efficiency.

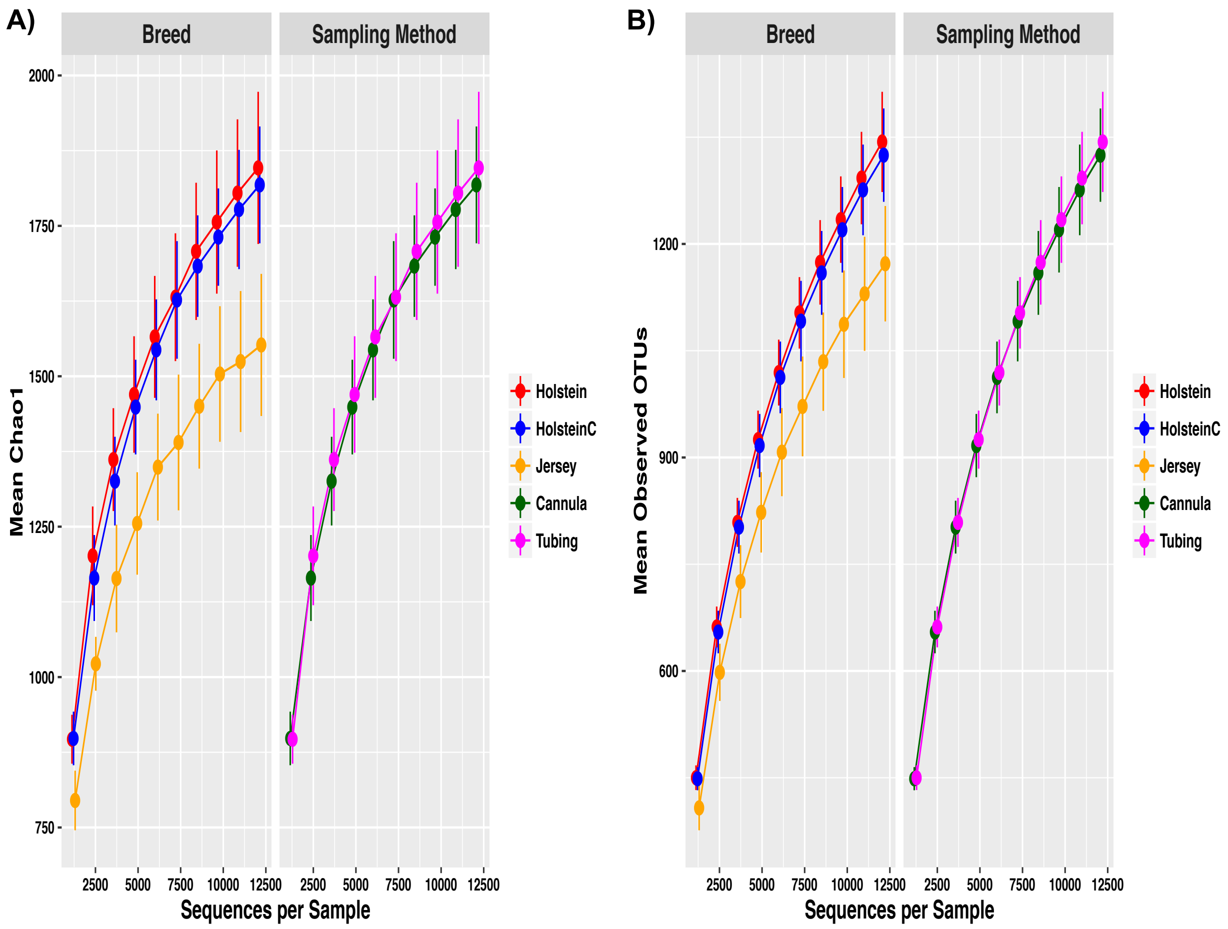

Supplementary Figure 3. Rarefaction curves at even depth (12,141 reads) across samples. **(A)** mean Chao1 estimate and **(B)** mean observed OTUs. The Holstein, Jersey, and tubing curves represent the rarefactions from samples collected via esophageal tubing while the HolsteinC and cannula curves represent the rarefactions from samples collected via the rumen cannula.

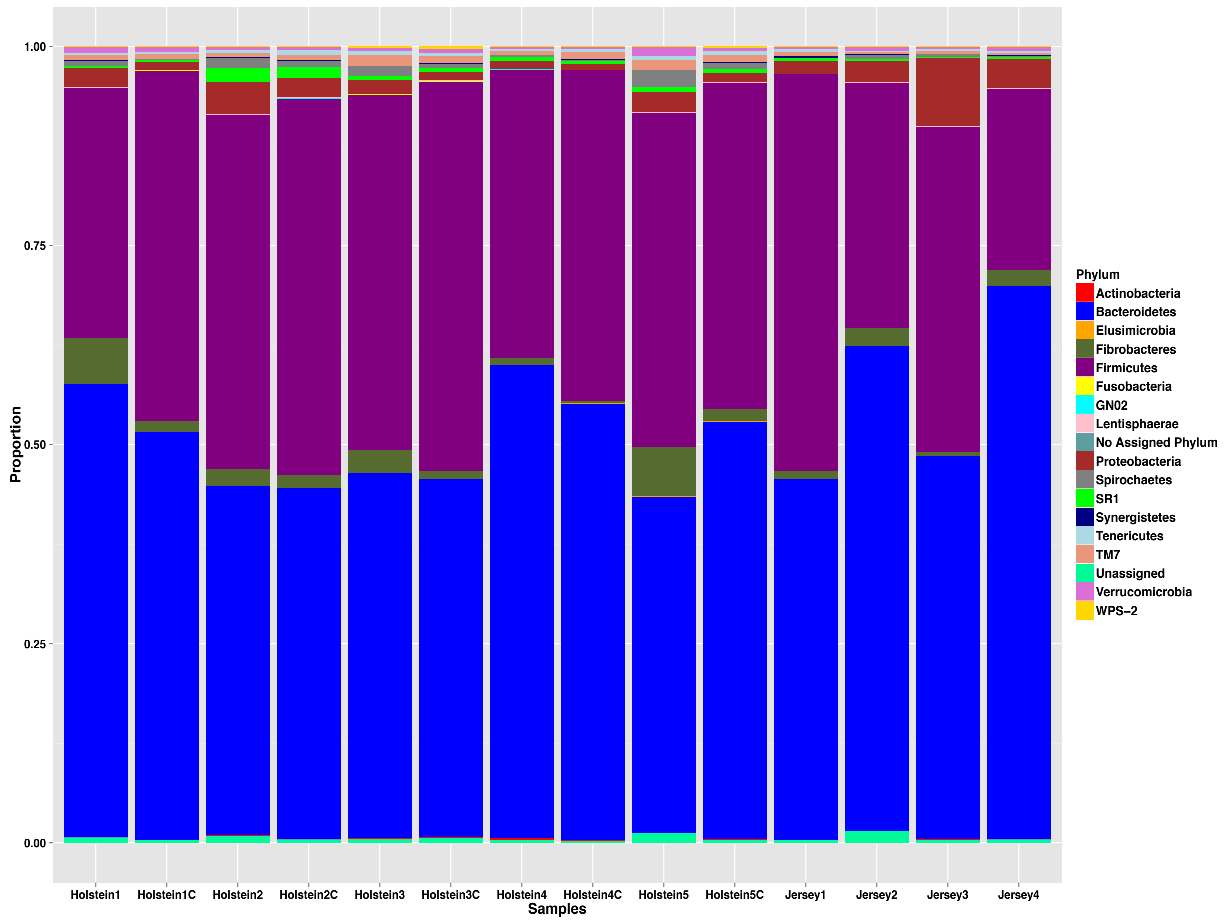

Supplementary Figure 4. Phylum level classification of the bacterial community composition across breeds and sampling methods. Samples labeled with a C at the end were collected via rumen cannula, whereas rest of the samples were collected via esophageal tubing.

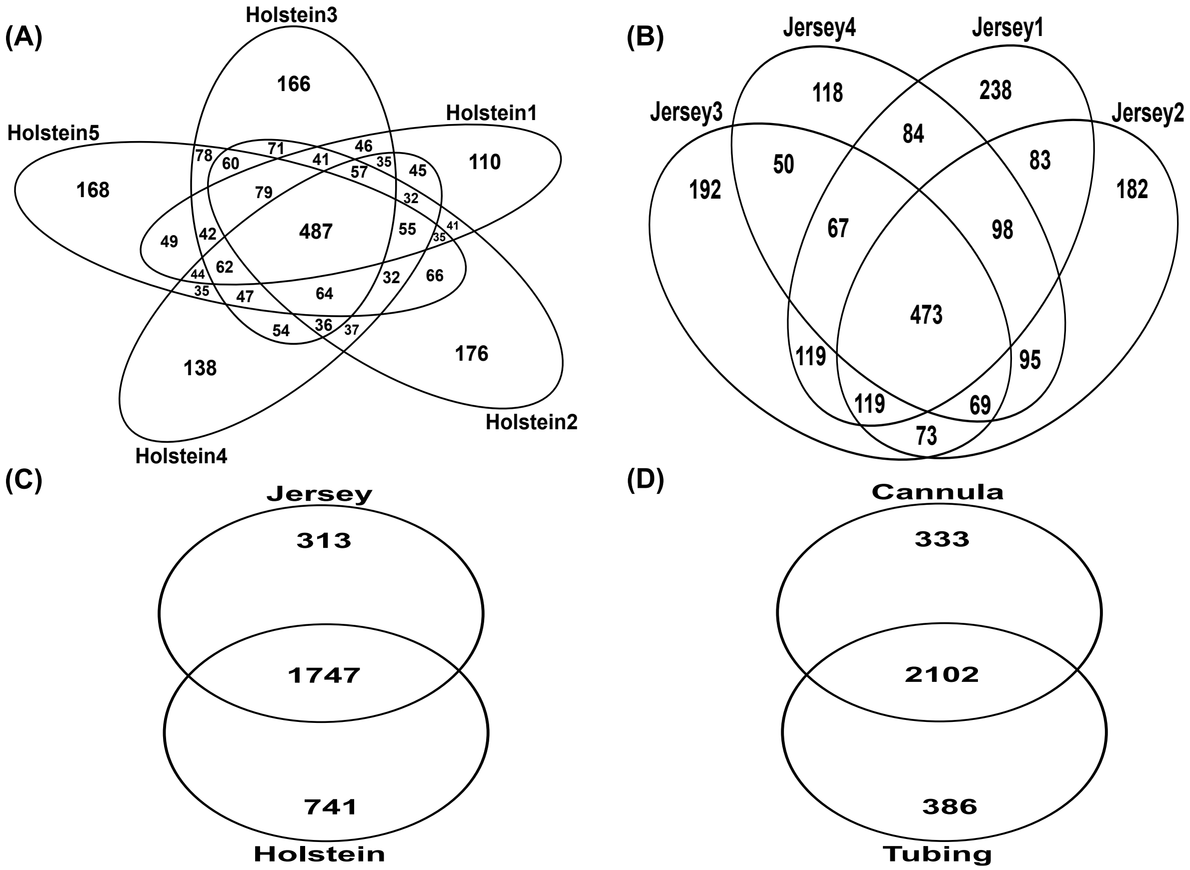

Supplementary Figure 5. Venn diagrams displaying the distribution of OTUs. (A) Holstein, **(B)** Jersey, **(C)** breeds, and **(D)** sampling methods.

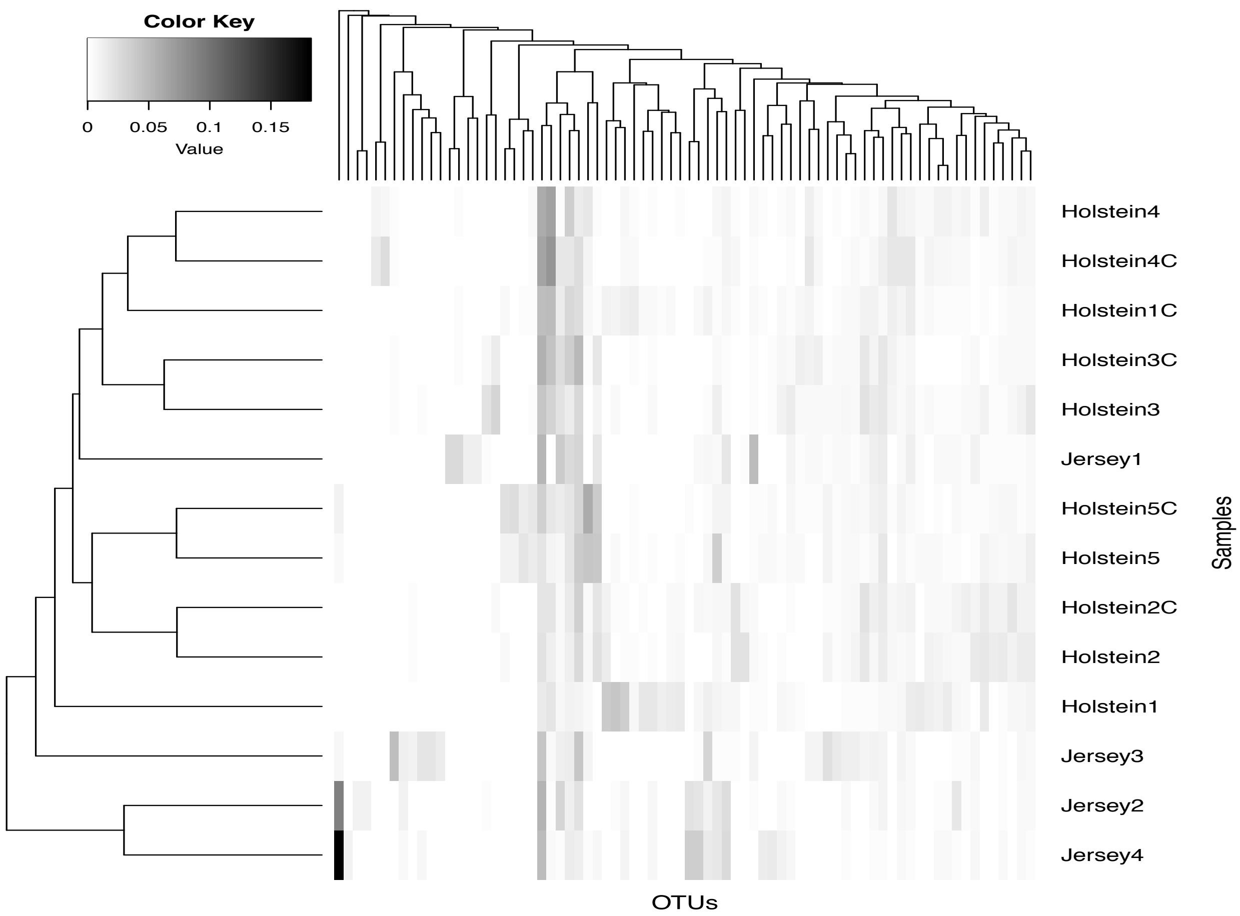

Supplementary Figure 6. Hierarchical clustering of all core OTUs identified in Holstein and Jersey cows with a minimum relative abundance of >1%. Samples labeled with a C at the end were collected via rumen cannula, whereas rest of the samples were collected via esophageal tubing.

Supplementary Table 1. Ingredient and chemical composition of the diet fed to all cows

Item	% DM
Ingredient	
Corn silage	31.7
Alfalfa hay	13.0
Brome hay	6.00
Ground corn	10.2
Soybean hulls	8.80
Soybean meal	3.00
Soy Pass[1]	4.00
Low-fat distillers dried grains with solubles	20.0
Megalac[2]	0.60
Limestone	1.00
Sodium bicarbonate	0.62
Dicalcium phosphate	0.60
Magnesium oxide	0.20
Salt	0.20
Trace minerals[3]	0.04
Vitamins premix[4]	0.04
Chemical composition[5]	
Crude protein	16.7
Neutral detergent fiber	39.0
NFC[6]	32.9
Ash	7.90
Crude fat	3.50
Net energy of lactation (mCal/kg)	1.61

[1]LignoTech, Overland Park, KS.
[2]Church & Dwight Co. Inc., Princeton, NJ.
[3]Contained 13.9% Ca, 0.03 % P, 0.42 % Mg, 0.20% K, 4.20% S, 0.08% Na, 0.03% Cl, 445 mg of Fe/kg, 60,021 mg of Zn/kg, 17,375 mg of Cu/kg, 43,470 mg of Mn/kg, 287 mg of Se/kg, 527 mg of Co/kg, and 870 mg of I/kg.
[4]Provided approximately 5,770 KIU of vitamin A/kg, 1,460 KIU of vitamin D/kg, and 46,400 KIU of vitamin E/kg.
[5]Estimations are based on CPM Dairy software (Boston et al., 2000)[b] using default values of the chemical composition of feeds.
[6]Non-fiber carbohydrates = 100 – (% NDF + % CP + % fat + % ash).

[b] Boston, R. C., D. G. Fox, C. J. Sniffen, R. Janczewski, R. Munsen, and W. Chalupa. 2000. *The conversion of a scientific model describing dairy cow nutrition and production to an industry tool: The CPM Dairy project, p 361-377. In* JP McNamara, J France, and D Beever (ed), Modelling Nutrient Utilization in Farm Animals, CABI Publishing, Oxford, UK.

Supplementary Table 2. Mean richness estimates for breeds and sampling methods

	Estimate[a]	
Item	Chao1	Observed OTUs
Breed		
Holstein	1,846*	1,343*
Jersey	1,552*	1,172*
Sampling method		
Esophageal tubing	1,846	1,343
Rumen cannula	1,818	1,325

* Within column for breed or sampling method, means differ ($P = 0.02$).

[a] Even sampling depth based on sample with the lowest number of sequences (12,141).
